# Serotonin depletion impairs both Pavlovian and instrumental reversal learning in healthy humans

**DOI:** 10.1038/s41380-021-01240-9

**Published:** 2021-08-24

**Authors:** Jonathan W. Kanen, Annemieke M. Apergis-Schoute, Robyn Yellowlees, Fréderique E. Arntz, Febe E. van der Flier, Annabel Price, Rudolf N. Cardinal, David M. Christmas, Luke Clark, Barbara J. Sahakian, Molly J. Crockett, Trevor W. Robbins

**Affiliations:** 1 Department of Psychology, University of Cambridge, Cambridge, UK; 2 Behavioural and Clinical Neuroscience Institute, University of Cambridge, Cambridge, UK; 3 Department of Neuroscience, Psychology, and Behaviour, University of Leicester, Leicester, UK; 4 Section of Eating Disorders, Department of Psychological Medicine, Institute of Psychiatry, Psychology and Neuroscience, King’s College London, London, UK; 5 Department of Psychology, Leiden University, Leiden, The Netherlands; 6 Department of Experimental Psychology and Helmholtz Institute, Faculty of Social and Behavioural Sciences, Utrecht University, Utrecht, The Netherlands; 7 Department of Psychiatry, University of Cambridge, Cambridge, UK; 8 Cambridgeshire and Peterborough NHS Foundation Trust, Cambridge, UK; 9 Department of Psychology and Djavad Mowafaghian Centre for Brain Health, University of British Columbia, Vancouver, BC Canada; 10 Department of Psychology, Yale University, New Haven, CT USA

**Keywords:** Psychology, Neuroscience

## Abstract

Serotonin is involved in updating responses to changing environmental circumstances. Optimising behaviour to maximise reward and minimise punishment may require shifting strategies upon encountering new situations. Likewise, autonomic responses to threats are critical for survival yet must be modified as danger shifts from one source to another. Whilst numerous psychiatric disorders are characterised by behavioural and autonomic inflexibility, few studies have examined the contribution of serotonin in humans. We modelled both processes, respectively, in two independent experiments (*N* = 97). Experiment 1 assessed instrumental (stimulus-response-outcome) reversal learning whereby individuals learned through trial and error which action was most optimal for obtaining reward or avoiding punishment initially, and the contingencies subsequently reversed serially. Experiment 2 examined Pavlovian (stimulus-outcome) reversal learning assessed by the skin conductance response: one innately threatening stimulus predicted receipt of an uncomfortable electric shock and another did not; these contingencies swapped in a reversal phase. Upon depleting the serotonin precursor tryptophan—in a double-blind randomised placebo-controlled design—healthy volunteers showed impairments in updating both actions and autonomic responses to reflect changing contingencies. Reversal deficits in each domain, furthermore, were correlated with the extent of tryptophan depletion. Initial Pavlovian conditioning, moreover, which involved innately threatening stimuli, was potentiated by depletion. These results translate findings in experimental animals to humans and have implications for the neurochemical basis of cognitive inflexibility.

## Introduction

Serotonin (5-HT; 5-hydroxytryptamine) is classically involved in responding to negative events, is increasingly recognised to be engaged in reward learning, and is important for adapting previously learned responses to reflect new environmental circumstances [1–10]. Whilst a unified framework for serotonin function remains elusive, considering how serotonin influences fundamental Pavlovian (stimulus-outcome) and instrumental (stimulus-response-outcome; operant) learning processes has the potential to make such an objective more tractable. Here, we studied healthy human volunteers to examine the effects of lowering serotonin synthesis on cognitive flexibility assessed by instrumental and Pavlovian reversal learning.

Reversal learning paradigms, whereby an initial contingency is learned and subsequently reverses, have revealed both Pavlovian and instrumental reversal learning deficits in obsessive-compulsive disorder (OCD), a prototypical disorder of cognitive inflexibility [11, 12]. Pavlovian reversal deficits have also been observed in post-traumatic stress disorder (PTSD) [13]. Instrumental reversal deficits, meanwhile, have been documented in schizophrenia [14], gambling disorder [15], and alcohol [16], methamphetamine, and cocaine use disorders [17]. Non-reversal aberrations in Pavlovian threat and safety learning have also been reported in OCD [18, 19], schizophrenia [20, 21], PTSD [22, 23], and other anxiety disorders [22, 24, 25].

Serotonergic dysfunction, at the same time, has been documented across diagnostic categories [26–33]. A recent meta-analysis, for example, showed decreased bioavailability of tryptophan, serotonin’s precursor, as well as a shift in the metabolism of tryptophan away from the serotonin biosynthesis pathway in major depressive disorder, bipolar disorder, and schizophrenia [30]. Moreover, post-mortem assessment of individuals with stimulant use disorder revealed decreased serotonin concentration [34] and serotonin transporter (SERT) density [35] in the orbitofrontal cortex (OFC). In OCD, reduced SERT in the OFC has been reported [36]. There is additionally an array of evidence from single-photon emission computed tomography (SPECT) and positron emission tomography (PET) of decreased SERT in various other brain regions in OCD, including in drug- naïve individuals, with some findings correlating with OCD symptom severity [28, 37–41]. In PTSD and panic disorder, several studies suggest an increased sensitivity of the 5-HT2C receptor as determined through pharmacological challenge [33, 42, 43]. Dysfunction of the 5-HT1A receptor, assessed by PET, has additionally been reported across PTSD, panic disorder, and social anxiety disorder [44–47].

Therefore, it is not surprising that drugs thought to boost serotonin transmission when given chronically—selective serotonin reuptake inhibitors (SSRIs)—are first line treatments for OCD [48], PTSD [49], and depression [50]. Schizophrenia is treated with drugs that modulate serotonin in addition to dopamine, such as risperidone, a non-selective serotonin 2A (5-HT2A) receptor antagonist [51].

Despite its broad clinical relevance, the preponderance of evidence on how serotonin impacts behavioural adaptation comes from studies of non-human animals [7, 52–56], whilst the role of serotonin in human threat and safety learning has received surprisingly little attention [57]. The experimental animal literature has focused on instrumental reversal learning, whereby a learned optimal behaviour (usually for obtaining food reward) becomes disadvantageous and another behavioural strategy needs to be adopted. Failure to adapt to new contingences is referred to as perseveration. A major advantage of this experimental approach is that similar paradigms, typically involving two choices, can be used across species. In rats, impairing serotonin function via neurotoxic depletion, chronic intermittent cold stress, or acute low dose SSRI (1 mg/kg citalopram) disrupted reversal learning [52, 56]. Enhancing serotonin function in rats via SSRI given acutely at higher doses (5 or 10 mg/kg citalopram), or administered repeatedly, enhanced reversal learning [52, 56]. There is robust evidence that intact serotonin function in the OFC is critical for reversal learning. Highly perseverative rats during reversal learning had reduced levels of 5-HT2A receptors and the serotonin metabolite 5-hydroxyindoleacetic acid (5-HIAA) in the OFC, and decreased expression of monoamine oxidase and tryptophan hydroxylase genes in the dorsal raphé nucleus (DRN) [53]. In marmoset monkeys, targeted neurotoxic serotonin depletion of the OFC via 5,7-dihydroxytryptamine (5,7-DHT), but not of the caudate nucleus, has consistently produced reversal deficits [54, 55, 58]. Depleting OFC serotonin has been proposed to promote stimulus-response associations over stimulus-response-reward goal-directed action [4, 59].

Whilst the effects of serotonin on Pavlovian threat reversal learning have not been studied in any species, to our knowledge, there is a body of work (mostly from non humans) indicating that serotonin influences threat conditioning processes [57], more commonly known as fear conditioning [60]. That Pavlovian threat conditioning can also be studied across species represents a major advantage. The directionality of effects is complex and can differ by serotonergic manipulation, experimental paradigm, dependent measure, stimulus, species, predictability, 5-HT receptor subtype, and brain region [57, 61–66]. That serotonin can have opposing effects to threats, however, is at the heart of an influential theoretical framework for understanding serotonin function [67].

Serotonin signalling is postulated to restrain physiological responses to proximal and innate threats (and thus inhibit panic), whilst promoting anticipatory anxiety for distal, learned threats [67]. Indeed, administration of the serotonin 2A/2C (5-HT2A, 5-HT2C) receptor antagonist ritanserin to healthy humans enhanced innate anxiety during simulated public speaking [68] yet reduced learned anticipatory anxiety during Pavlovian conditioning [69]. Consistent with this framework, serotonin depletion attenuated Pavlovian threat conditioning to inherently neutral cues and corresponding amygdala activity in healthy humans [70]. There are distinctions between circuits that respond to learned threats (e.g. neutral cues), predators (e.g. snakes or spiders), and aggressive conspecifics [71]. Serotonergic circuits can be engaged differentially by innate versus learned threats [72] and by the intensity of threat [73]. Secondary to our investigation of cognitive flexibility, we also addressed whether depleting serotonin would potentiate initial Pavlovian conditioning when employing innately threatening conditioned stimuli.

Here, we conducted two independent experiments employing acute tryptophan depletion (ATD) to determine the influence of serotonin on Pavlovian and instrumental reversal learning in healthy human volunteers. Depleting tryptophan, serotonin’s biosynthetic precursor, decreases serotonin synthesis and function [74–78]. Experiment 1 tested instrumental reversal learning. Individuals acquired an adaptive behaviour through trial and error learning, and the correct response subsequently changed multiple times, necessitating cessation of the previous action and performing a new behaviour. Experiment 2 examined reversal learning in the Pavlovian domain [11, 79]. In a Pavlovian threat conditioning procedure, participants were presented with two cues (threatening faces, i.e. signs of aggressive conspecifics), one of which was sometimes paired with an electric shock, while the other was not. A reversal phase followed, whereby the originally conditioned face became safe, and the initially safe face was newly paired with shock (Supplementary Fig. 1) [79]. To assess associative learning during Pavlovian conditioning and reversal, the skin conductance response (SCR) was used as a measure of (mostly sympathetic) autonomic nervous system responses [13, 79–83].

**Fig. 1:**
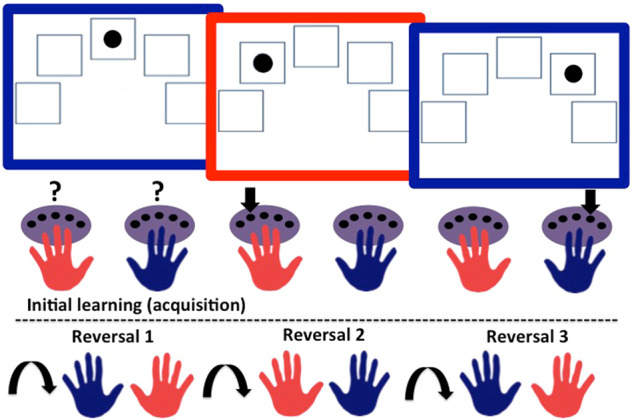
Experiment 1 task schematic. TOP: The three rectangles with coloured frames represent three example trials presented in the acquisition phase. Purple ovals symbolise the button boxes. Question marks signify the need to learn the correct hand-colour association by trial and error. Downward pointing arrows indicate the correct hand and button response for that trial. BOTTOM: Curved arrows signify the reversal of colour-hand contingencies, which occurred three times.

Impairments in human instrumental reversal learning following ATD have been difficult to detect to date [84–89]. Behaviour in previous studies, however, was not reinforced with motivationally salient feedback, which was instead more symbolic (e.g. ‘CORRECT’/‘WRONG’; ‘You win/lose 100 points’; higher or lower pitched tone). Consequently, there may not have been sufficient incentive to update or restrain action: any requirement for serotonin signalling to perform the task at hand may have been minimal enough to be unaffected by ATD [90]. Indeed, the depletion achieved by ATD is relatively mild in comparison with the profound depletion that is possible in experimental animals [52, 54, 55]. Given the importance of serotonin in processing both aversive [5, 91–93] and rewarding [1, 3, 7, 9] outcomes, we used an innovative task (Fig. 1) incorporating feedback that was markedly more salient than was used in previous reversal tasks [84–89]. Unlike previous human instrumental reversal learning tasks, the present paradigm allowed for the influence of serotonin on reward and punishment to be parsed. In this way, the reward condition in our paradigm paralleled the non-human animal studies whilst the punishment conditions extend the existing literature. ATD has produced different effects on goal-directed behaviour in healthy volunteers responding to obtain rewards versus avoid punishments [94], and therefore we predicted that effects of serotonin in the present experiment would depend on valence.

Prior studies that did not find a perseverative deficit following ATD employed largely probabilistic feedback [84–86] and a single reversal [85, 86]. Other ATD studies were used primarily to test observational reversal learning, where outcomes were not contingent on responses [95, 96], or higher order cognitive flexibility in the form of attentional set-shifting [87–89]. Similarly, genetic variation in the serotonin transporter was not associated with perseveration—our primary interest—but was related to inappropriate behavioural shifting after losses during probabilistic reversal [97]. Meanwhile, evidence of a perseverative deficit following neurotoxic serotonin depletion of the marmoset OFC, comes from a paradigm more similar to that employed in the present study [54]: serial reversals on a deterministic schedule (in the appetitive domain) were used, and a reversal deficit that emerged only beginning in the second reversal was found. We were therefore particularly interested in whether focusing on a later reversal phase may be key to uncovering perseveration following ATD in humans.

The aim of this study, therefore, was to address the following questions. In Experiment 1: Does ATD induce perseveration in instrumental reversal learning? Are deficits valence-dependent [94]? Do these effects emerge in a later reversal phase, and particularly when feedback is most salient? In Experiment 2: Does ATD impair Pavlovian reversal learning? And does ATD have a different effect on conditioning to threatening cues compared to neutral cues? In the instrumental domain, we hypothesised that ATD would lessen the impact of motivationally salient feedback to guide behaviour, resulting in a perseverative deficit. More specifically, we predicted that ATD would not impair reversal learning in the neutral feedback condition, but that serotonin would have a differential effect depending on salient rewarding and/or punishing feedback. In the Pavlovian domain, we predicted initial conditioning to innately threatening stimuli would be potentiated by ATD and that autonomic responses would not adapt flexibly to new contingencies following reversal. Instrumental and Pavlovian reversal learning deficits following serotonin depletion would collectively point to a requirement of serotonin for integrating new information about reinforcement contingencies, which is fundamental to daily life and well-being.

## Materials and methods

### Acute tryptophan depletion

Healthy volunteers were assigned to receive ATD or placebo, in a randomised, double-blind, between-groups design. The ATD group consumed a drink containing the essential amino acids less tryptophan, whereas the placebo drink was identical other than it included tryptophan (see Supplementary Information for details). Blood samples were taken to verify depletion.

### EXPERIMENT 1

#### Participants

Sixty-nine healthy participants (mean age 24.28, 36 males) completed the deterministic reversal learning task and were included in the final analysis. One male participant in the depletion group was excluded because he admitted to responding randomly later in the task. Participants were screened to be medically healthy and free from any psychiatric conditions, determined by the Mini International Neuropsychiatric Interview [98]. Individuals who reported having a first-degree relative (parent or sibling) with a psychiatric disorder were excluded upon screening as well (see Supplementary Information for further screening criteria). Volunteers provided informed consent before the study and were paid for their participation. Groups did not differ in age, years of education, trait impulsivity, or in baseline depressive and obsessive-compulsive symptoms, shown in Supplementary Table 1.

#### General procedure

The protocol was approved by the Cambridge Central NHS Research Ethics Committee (Reference # 16/EE/0101). The study took place at the National Institute for Health Research/Wellcome Trust Clinical Research Facility at Addenbrooke’s Hospital in Cambridge, England. Participants arrived in the morning having fasted for at least 9 h prior, gave a blood sample, and ingested either the placebo or ATD drink. To assess mood and other feelings including alertness, we used a 16-item visual analogue scale (VAS) at the beginning, middle, and end of the day-long testing session. In the afternoon participants completed the deterministic reversal learning task, along with several other tasks reported elsewhere [65, 99, 100].

#### Instrumental reversal learning task

The task used in Experiment 1, developed by Apergis-Schoute [101], is depicted in Fig. 1. As an incentive, participants were told that depending on how well they performed the task, they could win a bonus on their compensation for taking part in the study. In reality, everyone received a small bonus. The instrumental reversal paradigm was designed to increase task demands and thus difficulty in comparison with previous reversal tasks [84–89], by including serial reversals, salient feedback, and necessitating specific hand and finger response mappings to stimuli. It had three reversals and a deterministic schedule. Responses were entered via one of two ‘button boxes’ with either the left or right hand, see Fig. 1. On each trial, the computer screen was framed by a specific colour and displayed five boxes corresponding to five buttons on each button box, one button per finger. The colour indicated the correct hand to respond with, and a black dot inside one of the five boxes on the screen indicated which finger to respond with, depicted in Fig. 1. Participants were told they needed to learn the colour-hand association by trial and error and that the association would change multiple times within a run. A correct response required responding both with the correct finger and the correct hand. A run consisted of four blocks of 20 trials each: an acquisition block where the initial contingency was established followed by three reversal blocks. The reinforcement schedule was deterministic: the correct option led to positive feedback on 100% of trials, whilst the incorrect response led to negative feedback on 100% of trials. Trial order was randomised. There were four runs in random order, and each contained a unique pair of colours framing the screen which was counterbalanced. All runs contained the same visual feedback cartoon stimuli (Supplementary Fig. 2): a smiling face with ‘two thumbs up’ for correct responses, a face showing disappointment and a ‘thumbs down’ when incorrect, and an analogue alarm clock with a frown if a response was not entered within the allotted time. The salience and valence of feedback across runs was varied using the presence or absence of prominent auditory stimuli. The primary run of interest had the most salient auditory feedback: responding correctly to one colour resulted in reward in the form of a prominent ‘cha-ching’ (slot machine) sound, whilst correct responses to the other colour prevented (avoided) the occurrence of an aversive buzzer noise (reward-punishment run). There was also a reward-neutral run where a correct response to one colour frame resulted in the reward auditory feedback whereas responding correctly or incorrectly to the other colour resulted only in visual (neutral) but no auditory feedback. In the punishment-neutral run, incorrect responses to one colour frame were punished with the buzzer noise whereas correct or incorrect responses to the other colour resulted only in visual feedback (neutral). Finally, the task contained a neutral-neutral condition where no auditory feedback was provided and only visual feedback via cartoons was presented.

The experiment began with three training phases, each of which required making correct responses on at least 80% of trials to advance to the next stage otherwise the phase would be repeated. The first was self-paced and served to familiarise participants with responding using the button boxes. In the first training phase only, ‘LEFT’ or ‘RIGHT’ was displayed on each trial to instruct the correct hand to use. There was a time limit in the second (short) and third (longer) training phases and participants were told to respond as quickly and accurately as possible. The time window to make responses during the actual experiment was automatically calibrated to each person based on their reaction times during the final practice phase. The task was programmed in E-Prime 2.0 Professional. The primary dependent measure was trials to criterion, as used in serotonin depletion studies in marmoset monkeys [54, 55], which we aimed to translate to humans here. The criterion was defined as making four consecutive correct responses.

### EXPERIMENT 2

#### Participants

Thirty healthy volunteers (mean age 25.44, 17 females) completed the Pavlovian threat reversal task. Of these, two (1 female) were deemed ‘non-responders’ for an undetectable SCR and were thus excluded based on the following criteria: having SCR recordings with a magnitude of less than 0.05 microsiemens (μS) on fewer than half of the CS+ trials during the acquisition phase. Most studies define ‘non-responders’ based on CS responses; however, see [102] for a discussion. All participants provided written informed consent and were financially compensated. The study was approved by the Cambridgeshire 2 Research Ethics Committee (Reference # 09/H0308/51). Participants were eligible if they did not have a personal or family history of major depressive disorder, bipolar disorder, or any other psychiatric illness. Groups did not differ in age, years of education, trait impulsivity, or in baseline depressive symptoms, shown in Supplementary Table 2.

#### General procedure

Participants were assigned to receive either placebo or the tryptophan-depleting drink in a randomised, double-blind design (16 received depletion). Blood samples were collected at baseline and before the task to verify tryptophan depletion. Participants completed questionnaires including one assessing self-reported mood state. Data were collected inside of a functional magnetic resonance imaging (fMRI) scanner, but the fMRI data are not reported here. Participants returned for a second session, where they received the other drink condition and also completed different computerised tasks, results of which have been published elsewhere [103, 104]. It is important that participants are naïve to conditioning paradigms, and therefore the data reported here were acquired in the first of two testing sessions spaced at least 1 week apart.

#### Conditioning procedure

The task [11] is depicted in Supplementary Fig. 1 and had two phases: acquisition and reversal. Two faces (face A and B) were presented in each phase, for 4 s each with an inter-trial interval of 12 s [79]. The face images were selected from the Ekman series [105]. Participants chose a shock level that they felt was uncomfortable but not painful. In the acquisition phase, face A was presented 16 times without a shock (conditioned stimulus plus; CS+) and coterminated with a 200 ms shock (unconditioned stimulus; US) on an additional eight trials (CS + US) that were spread throughout the acquisition phase, while face B was presented 16 times and never paired with shock (CS−). In the reversal phase the faces were presented again only the contingencies swapped: face A was presented for 16 trials and was no longer paired with a shock (new CS−), while face B was newly paired with a shock on 8 trials amidst an additional 16 unreinforced trials (new CS+). Trials were pseudorandomised and designation of face A and B was counterbalanced. Reversal was unsignaled and immediately followed acquisition without a break. SCR was the dependent measure. The primary focus was the SCR to unreinforced trials, to avoid contamination by the shock itself. SCRs were defined as the base-to-peak difference during a 7 s interval beginning 0.5 s after stimulus onset. SCRs were normalised for each individual participant by dividing values from each trial by the peak amplitude.

#### Multiple comparisons

Correction for multiple comparisons where relevant, was conducted using the Benjamini-Hochberg procedure [106]. The critical value for false discovery rate was set a priori [107] at *q* = 0.15 [108, 109].

## Results

### EXPERIMENT 1

#### Group-level instrumental learning

##### Omnibus analysis

**Fig. 2:**
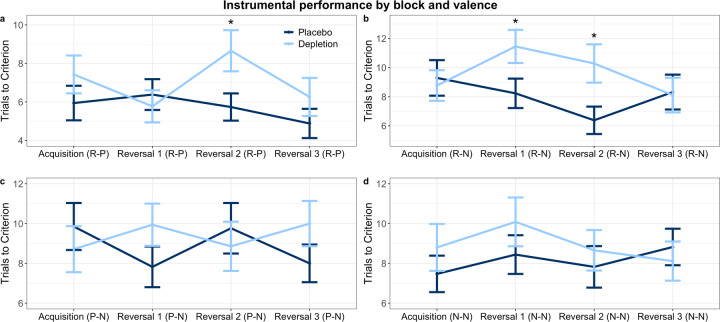
Instrumental reversal learning performance by block. More trials to criterion signifies worse performance. Asterisks represent significance at *p* < 0.05. Error bars represent ±1 standard error of the mean. **a** Impairment in Reversal 2 of the reward-punishment condition (R-P). **b** Impairments in Reversals 1 and 2 of the reward-neutral condition (R-N). **c** No differences in instrumental performance in the punishment-neutral condition (P–N). **d** No differences in instrumental performance in the neutral-neutral condition (N–N).

Instrumental reversal learning was impaired following ATD, and the core deficits are displayed in Fig. 2. First, omnibus repeated measures analysis of variance (ANOVA) was performed across all valence conditions and blocks. In the most salient condition participants had to make separate responses to obtain reward and avoid punishment (reward-punishment; see Methods). The other conditions incorporated either only neutral feedback (neutral-neutral), or neutral feedback with reward (reward-neutral) or punishment (punishment-neutral). The dependent measure for all analyses was trials to criterion (see Methods). Reaction time data are presented in the Supplementary Information. The omnibus ANOVA, with serotonin status (placebo, depletion) as a between-subjects factor, valence (reward-punishment, reward-neutral, punishment-neutral, neutral-neutral) and block (acquisition, reversal 1, reversal 2, reversal 3) as within-subjects factors, revealed a significant serotonin × valence × block interaction (*F*_(9,603)_ = 2.024, *p* = 0.035, *η*_p_^2^ = 0.029). There was no main effect of serotonin status (*F*_(1,67)_ = 1.869, *p* = 0.176, *η*_p_^2^ = 0.027).

##### Acquisition learning

Next we verified that this effect was not driven by acquisition learning. Indeed, ATD had no effect on initial discrimination learning in the reward-punishment condition (*t*_(67)_ = 1.115, *p* = 0.269, *d* = 0.268), reward-neutral (*t*_(67)_ = −0.325, *p* = 0.746, *d* = −0.078), punishment-neutral (*t*_(67)_ = −0.688, *p* = 0.494, *d* = −0.166) or neutral-neutral conditions (*t*_(64)_ = 0.891, *p* = 0.376, *d* = 0.214), shown in Fig. 2.

##### Reversal blocks

Results are shown in Fig. 2. To assess the nature of the reversal learning deficit, the significant three-way interaction was followed up with *t* tests in a sequence guided by two key a priori hypotheses. First, serotonin signalling is particularly engaged when responding to motivationally salient feedback [90], and therefore a reversal learning deficit should be most likely in the highest salience condition (reward-punishment). Second, serotonin depletion in the marmoset monkey OFC has been shown to induce the most pronounced instrumental reversal learning deficit in the second reversal block, without impacting the initial reversal [54]. The first follow-up test of reversal learning, therefore, assessed the second reversal of the most salient condition (reward-punishment) and indeed revealed a deficit: participants under ATD required more trials to criterion than on placebo (*t*_(59)_ = 2.281, *p* = 0.026, *d* = 0.546). We then tested whether the effect in the second reversal was present in the other, less salient, conditions. There was a significant deficit under ATD in the reward-neutral condition (*t*_(61)_ = 2.413, *p* = 0.019, *d* = 0.578), and not in the punishment-neutral (*t*_(67)_ = −0.512, *p* = 0.61, *d* = −0.123) or neutral-neutral (*t*_(67)_ = 0.572, *p* = 0.569, *d* = 0.138) conditions. Next we tested whether a deficit was present in the first reversal. Individuals under depletion required more trials to criterion in the reward-neutral condition (*t*_(67)_ = 2.113, *p* = 0.038, *d* = 0.509), but not in the reward-punishment (*t*_(67)_ = −0.528, *p* = 0.599, *d* = −0.127), punishment-neutral (*t*_(67)_ = 1.439, *p* = 0.155, *d* = 0.346), or neutral-neutral (*t*_(64)_ = 1.051, *p* = 0.297, *d* = 0.252) conditions. Finally, we assessed whether there was any deficit in the last reversal block. Performance was not impaired in the final reversal phase in the reward-punishment (*t*_(67)_ = 1.097, *p* = 0.277, *d* = 0.264), reward-neutral (*t*_(67)_ = −0.124, *p* = 0.902, *d* = −0.030), punishment-neutral (*t*_(67)_ = 1.348, *p* = 0.182, *d* = 0.325), or neutral-neutral (*t*_(67)_ = −0.526, *p* = 0.601, *d* = −0.127) conditions. The key deficits, from the reward-punishment and reward-neutral conditions identified in the second reversal block, additionally survived the Benjamini-Hochberg procedure (see Methods), for 12 comparisons (four valence conditions and three reversals), and were therefore the primary drivers of the serotonin × valence × block interaction.

#### Relationship between instrumental reversal deficits and extent of depletion

**Fig. 3:**
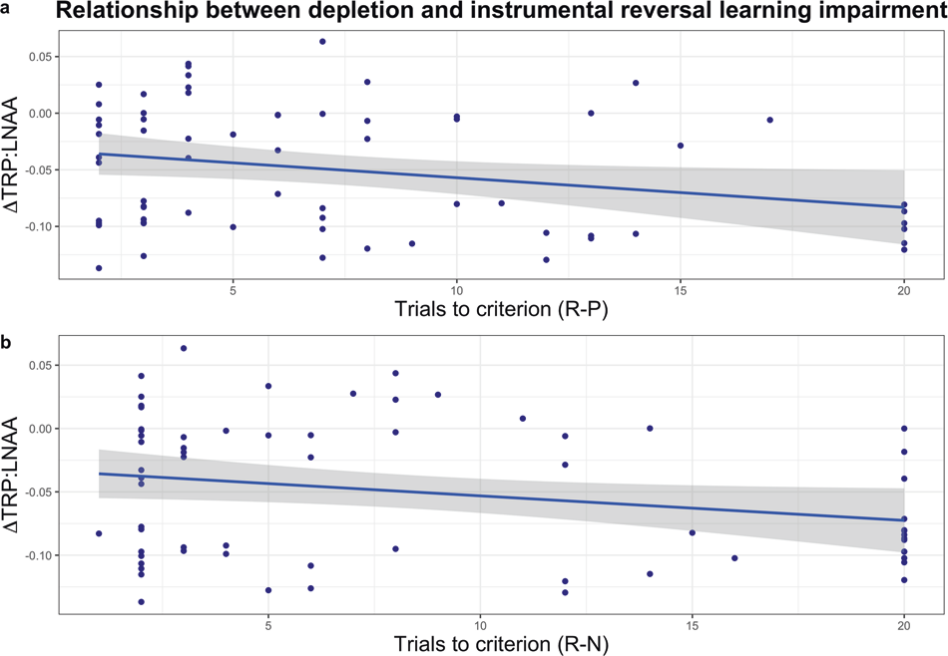
Relationship between extent of depletion and instrumental reversal learning performance. **a** Reward-punishment (R-P) condition. **b** Reward-neutral (R-N) condition. Both correlations were significant. ΔTRP:LNAA is the change in the ratio of tryptophan to large neutral amino acids; 0 indicates no change. A greater decrease (post-depletion minus pre-depletion blood plasma results) in the TRP:LNAA ratio indicates a more extensive depletion (more negative *y*-axis values). Reversal learning is indexed here as the number of trials to criterion in the second reversal block. Increasing *x*-axis values represent more trials to criterion and thus worse reversal performance. Shading indicates ±1 standard error (SE).

More pronounced depletion was significantly correlated with the key reversal deficits, shown in Fig. 3. To further substantiate the deficits observed upon depletion, correlation analyses between behaviour and individual subject plasma samples were conducted. First, this was performed for behaviour in the second reversal block during both the reward-punishment and reward-neutral conditions, where significant deficits were found at the group level. Indeed, greater extent of depletion was significantly correlated with the magnitude of these key reversal impairments: more pronounced depletion was related to worse performance in both the reward-punishment condition (*r*_(66)_ = −0.266, *p* = 0.031) and the reward-neutral condition (*r*_(66)_ = −0.25, *p* = 0.043). These results are displayed in Fig. 3a, b, respectively. The other observed behavioural impairment, from the first reversal in the reward-neutral condition, was also significantly correlated with the extent of depletion (*r*_(66)_ = −0.311, *p* = 0.011).

#### Blood analysis and mood

Robust tryptophan depletion was achieved, as verified by plasma samples (*t*_(64)_ = −18.725, *p* = 1.161 × 10^−27^, *d* = −4.610) using the ΔTRP:LNAA (change, from baseline to approximately 4.5 h after drink administration [110], in the ratio of tryptophan to large neutral amino acids [75] (see Supplementary Information). The mean ΔTRP:LNAA was −0.000023 (standard error of the mean, SEM = 0.004480) in the placebo group and −0.100244 (SEM = 0.002928) in the depletion group. Plasma levels were unavailable for three participants: one due to an error in the centrifugation and freezing procedure, and two due to difficulty with venepuncture. Self-reported mood assessed using a VAS, available for 63 participants, was unaffected by ATD (*p* > 0.05).

### EXPERIMENT 2

#### Omnibus analysis

**Fig. 4:**
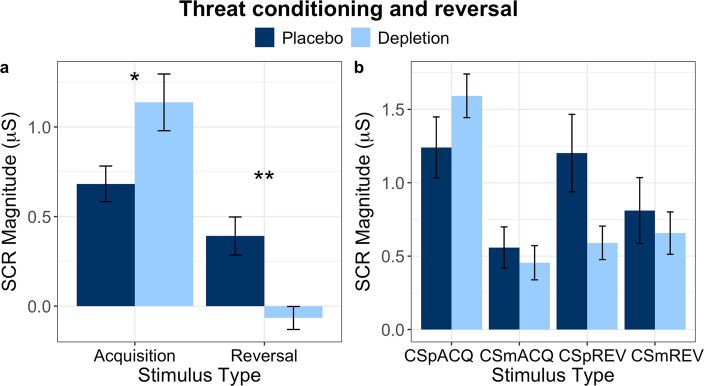
Pavlovian acquisition and reversal SCR data (Experiment 2), visualised in two different ways. Error bars represent ±1 standard error (SE). **a** Difference scores of CS+ minus CS−, indicative of the extent of discrimination learning to the two stimuli. Asterisks (*) indicate significance at *p* < 0.05; double asterisks (**) denote significance at *p* < 0.01. **b** All stimuli displayed separately. CSpACQ = (initial) CS+ during acquisition; CSmACQ = (initial) CS− during acquisition; CSpREV = (new) CS+ during reversal; CSmREV = (new) CS– during reversal.

SCR data are displayed in Fig. 4a, b. First, we performed an omnibus analysis to determine whether SCR to the two stimuli across both phases was affected by ATD. Repeated measures ANOVA, with serotonin status (placebo, depletion) as a between-subjects factor and phase (acquisition, reversal) and stimulus (CS+, CS−) as within-subjects factors revealed a significant three-way serotonin × phase × stimulus interaction (*F*_(1,26)_ = 17.604, *p* = 0.00028, *η*_p_^2^ = 0.404). Additionally, there were significant two-way interactions between phase × stimulus (*F*_(1,26)_ = 47.225, *p* = 2.7 × 10^−7^, *η*_p_^2^ = 0.645) and serotonin × phase (*F*_(1,26)_ = 11.258, *p* = 0.002, *η*_p_^2^ = 0.302). There was a main effect of stimulus (*F*_(1,26)_ = 73.410, *p* = 4.77 × 10^−9^, *η*_p_^2^ = 0.738), no main effect of phase (*F*_(1,26)_ = 3.756, *p* = 0.064, *η*_p_^2^ = 0.126), and no serotonin × stimulus interaction (*F*_(1,26)_ = 0.0001, *p* = 0.992, *η*_p_^2^ = 4 × 10^−6^). There was no main effect of serotonin status (*F*_(1,26)_ = 0.374, *p* = 0.546, *η*_p_^2^ = 0.014). Pavlovian reversal deficits were confirmed in an additional ANOVA. Two delta scores were calculated [111]: new CS− minus old CS+, and new CS+ minus old CS−. The two delta scores were entered into the ANOVA as a two-level within-subject factor, and the between-subjects factor was serotonin status (placebo, depletion). This revealed a main effect of serotonin status (*F*_(1,26)_ = 11.258, *p* = 0.002, *η*_p_^2^ = 0.302), an effect of stimulus (*F*_(1,26)_ = 73.410, *p* = 4.77 × 10^−9^, *η*_p_^2^ = 0.738), and no serotonin × stimulus interaction (*F*_(1,26)_ = 1.03 × 10^−4^, *p* = 0.992, *η*_p_^2^ = 4 × 10^−6^).

#### Acquisition of conditioning

Conditioning data are displayed in Fig. 4a, b. Differential conditioning (CS+ versus CS−) was attained in both the placebo and ATD groups (follow-up paired *t* tests: *t*_(11)_ = 6.866, *p* = 0.000027, *d* = 1.982, for placebo; *t*_(15)_ = 7.181, *p* = 0.000003, *d* = 1.795, for depletion). Conditioning was significantly stronger following depletion compared to the placebo group: we calculated a difference score of CS+ minus CS− for each group, and the magnitude of the CS+ relative to the CS− was significantly greater in the ATD group (*t*_(26)_ = −2.245, *p* = 0.034, *d* = −0.857).

#### Reversal of conditioning

The reversal learning results are depicted in Fig. 4a, b. During the reversal phase, follow-up *t* tests indicated the placebo group successfully conditioned to the new CS+ (*t*_(11)_ = 3.684, *p* = 0.004, *d* = 1.064). The depletion group, however, did not show discrimination between the new CS+ and the new CS− (*t*_(15)_ = −1.031, *p* = 0.319, *d* = −0.258), indicating a reversal learning impairment. Comparing the difference score during the reversal phase (new CS+ minus new CS−) between placebo and ATD also confirmed reversal learning was impaired (*t*_(26)_ = 3.880, *p* = 0.001, *d* = 1.482).

#### Changes in physiological responses to stimuli across phase: old versus new

Follow-up paired *t* tests showed that responding to the initial CS+ extinguished upon reversal both within the placebo group (initial CS+ versus new CS−; *t*_(11)_ = 2.799, *p* = 0.017, d = 0.808) and under ATD (*t*_(15)_ = 6.402, *p* = 0.000012, *d* = 1.6). SCR to the initial CS−﻿ increased upon reversal in the placebo group (*t*_(11)_ = −4.172, *p* = 0.002, *d* = −1.204) but critically, there was no difference in SCR to the initial CS− in acquisition compared to the new CS+ (old CS−) in reversal after ATD (*t*_(15)_ = −1.370, *p* = 0.191, *d* = −0.342). The reversal impairment following ATD was driven by a failure to assign new aversive value, whereas safety learning upon reversal was intact.

#### Correlations between Pavlovian measures and extent of depletion

**Fig. 5:**
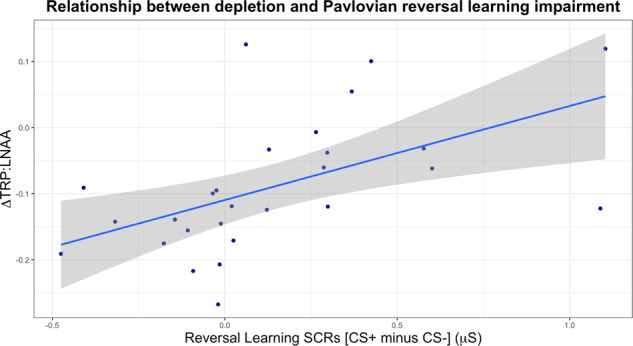
Experiment 2. Relationship between extent of depletion and degree of Pavlovian reversal learning impairment. The correlation was significant. ΔTRP:LNAA is the change in the ratio of tryptophan to large neutral amino acids; *y* = 0 indicates no change. A greater change (post-depletion blood minus pre-depletion results) in the TRP:LNAA ratio indicates a more extensive depletion (more negative *y*-axis values). Reversal learning is indexed here as the difference score between CS+ and CS− in the reversal phase. Increasing *x*-axis values represent better discrimination learning assessed by SCR between the CS+ and CS− in the reversal phase (i.e. better reversal learning). Shading indicates ±1 standard error (SE).

Next we tested whether the extent of depletion, as assessed via plasma samples, was related to our measures of Pavlovian threat conditioning and reversal. Extent of depletion was not correlated with the magnitude of the SCR difference score in the acquisition phase (*r*_(27)_ = 0.210, *p* = 0.294); however, there was a highly significant correlation between greater depletion and a more pronounced reversal learning deficit (*r*_(27)_ = −0.536, *p* = 0.004), depicted in Fig. 5. The Pavlovian reversal learning deficit was indexed by SCR to the CS+ minus the CS− in the reversal phase.

#### Unconditioned responses

Unconditioned responses [URs] (SCR to the shock itself) were unaffected by ATD. The peak UR for each subject was extracted from each phase (acquisition and reversal). Repeated measures ANOVA with serotonin status (placebo, depletion) as a between-subjects factor and phase (acquisition, reversal) as a within-subjects factor revealed no main effect of serotonin status on URs (*F*_(1,26)_ = 0.015, *p* = 0.904, *η*_p_^2^ = 0.001) and no serotonin × phase interaction (*F*_(1,26)_ = 1.137, *p* = 0.296, *η*_p_^2^ = 0.042). There was a significant effect of phase (*F*_(1,26)_ = 9.311, *p* = 0.005, *η*_p_^2^ = 0.264), such that URs were lower during the reversal phase than in the acquisition phase. The extent of depletion was not correlated with URs during acquisition (*r*_(27)_ = 0.04, *p* = 0.843) or reversal (*r*_(27)_ = −0.14, *p* = 0.485).

#### Blood analysis and mood

Robust depletion was also achieved in Experiment 2 (*t*_(17)_ = −4.907, *p* = 0.000132, *d* = −2.008). The mean ΔTRP:LNAA was 0.009225 (SEM = 0.025939) in the placebo group and 0.153429 (SEM = 0.013812) in the depletion group. Blood results from one participant were unavailable. Mood, assessed with the positive and negative affect schedule [112] after depletion had taken effect, was unaffected: there was no difference between serotonin status for positive (*p* > 0.05) or negative affect (*p* > 0.05).

## Discussion

We have provided convergent evidence from two independent experiments that serotonin depletion effected by acute dietary tryptophan depletion impairs human reversal learning in both the instrumental and Pavlovian domains (Experiments 1 and 2, respectively). The magnitude of the instrumental and Pavlovian reversal deficits, moreover, were both correlated with the extent of depletion assessed by plasma samples. Both the human instrumental and Pavlovian results are further strengthened by their consistency with studies of experimental animals following neurotoxic serotonin depletion [52, 54–56]. Remarkably, in rats, marmosets, and humans, the effect of serotonin depletion in the instrumental domain emerged most consistently upon the second reversal of contingencies [52, 54, 55]. Pavlovian extinction, meanwhile, was intact following serotonin depletion in humans, which is also consistent with data from marmosets following OFC serotonin depletion: (instrumental) extinction was unimpaired [113]. At the same time, initial Pavlovian conditioning to innately threatening cues was enhanced under serotonin depletion. Mood was unaffected, in line with the ATD literature in healthy humans [75, 114, 115].

Perseverative deficits in human instrumental reversal learning following ATD have not been easily captured to date [84–86], possibly owing in part to ATD inducing a transient and relatively mild depletion in comparison [116] with the profound depletion that is possible in experimental animals using 5,7-DHT [52, 54, 55, 113]. These ATD studies employed largely probabilistic feedback [84–86], with a single reversal [85, 86], and non-salient feedback [84–86]. The innovative instrumental task used here was unique in that it incorporated highly salient feedback, multiple reversals on a deterministic schedule, and increased cognitive load. The deterministic schedule with multiple reversals, in particular, aligns with the design of marmoset studies that have provided quintessential evidence that OFC serotonin depletion induces perseveration [54, 55].

Whilst the instrumental deficits on both the most salient (reward and punishment) and reward-only, but not punishment-only condition, as reported here, may at first seem surprising given the well-established role of serotonin in aversive processing [4, 5], this indeed aligns with the literature across species: the key marmoset studies on serotonin depletion and perseveration were conducted in the appetitive domain [54, 55, 113], and human ATD affected the appetitive but not aversive domain in a 4-choice probabilistic task on which computational modelling also revealed enhanced perseveration [9]. The depletion group here, nonetheless, performed worse numerically in reversals 1 and 3 (Fig. 2c) during the punishment-only condition.

There are several possible explanations for the instrumental reversal deficits observed following ATD. A marmoset study, employing reinforcement that most closely resembles the reward-neutral condition in the present study, and designed to interrogate the nature of the deficit that emerged upon the second reversal of contingencies [54], indicated that the reversal impairment following 5,7-DHT in OFC was due to an inability to disengage from the previously rewarded stimulus, rather than a failure to re-engage with the previously incorrect stimulus (learned avoidance) or reduced proactive interference [55]. When the subject arrives at the second reversal, two sets of competing associations have been experienced previously—the original and the reversed contingencies. While less likely applicable to the reward-neutral data, it remains possible that the deficit observed in the reward-punishment condition—a reinforcement structure not examined in marmosets [54, 55]—is related to an attenuation of proactive interference following ATD that ordinarily (under placebo conditions) biases responding towards the original association. While it is unclear why deficits were not observed in the third reversal, it is possible the aforementioned underpinning effects were short-lived.

The Pavlovian reversal findings reported here resemble those reported in OCD [11] and healthy humans under stress [117], and align with other studies of serotonin in rats, monkeys, and humans [56, 69, 113]. The Pavlovian reversal deficit in OCD, indexed by SCR on an identical paradigm, was explained by dysfunctional activity in the ventromedial prefrontal cortex (vmPFC), which receives rich serotonergic innervation [118]. Likewise, using SCR and a similar design to that used here (but with neutral cues), upon reversal, stress also attenuated the acquisition of threat responses to the newly threatening (previously safe) stimulus, while leaving extinction learning to the previously threatening cue intact [117]. The Pavlovian reversal deficits after serotonin depletion, in the present study, and after stress [117], are furthermore consistent with the finding that under 5-HT2A/C receptor blockade, a CS− (presented during habituation) failed to acquire aversive value during subsequent threat conditioning [69]. These parallels are striking, and are consonant with data from rats: stress, and separately serotonin depletion, produced comparable deficits in (instrumental) reversal learning [56]. Serotonin release in rats during behavioural testing, moreover, was reduced by stress, and an SSRI given acutely ameliorated the detrimental effect of stress on reversal learning [56]. The deleterious effects of serotonin depletion and stress on reversal learning can be interpreted as a selective impairment in integrating new information about a change in reinforcement contingencies, needed to update the representation of aversive value appropriately [117].

There are a number of theoretical and empirical considerations that can help link the instrumental and Pavlovian results. Whilst we do not know the neural locus of the present reversal impairments following ATD, work in the instrumental domain from experimental animals [54, 55, 113] and individuals with OCD [12, 119] enables us to highlight the OFC. The Pavlovian reversal data from OCD, at the same time, point to the vmPFC [11]. Indeed, damage subsuming the human OFC and vmPFC (which may include medial OFC structures such as area 14) impairs flexible stimulus-outcome learning and value-guided choice consistency, which may reflect disrupted integration of values on the basis of recalled outcomes [120]. Rhesus monkeys with lesions to the anterior cingulate cortex (ACC), meanwhile, showed impaired reversal of both action-outcome and stimulus-outcome contingencies [121]. Furthermore, there is evidence that ATD reduces Pavlovian influences over instrumental action in healthy humans, including in a Pavlovian-to-instrumental transfer (PIT) test, albeit selectively under conditions of punishment [92, 93]. Depleting OFC serotonin has been proposed to remove descending inhibitory mechanisms that ordinarily bias away from aversive processing (engaging with negative stimuli or outcomes), which would also account for the promotion of stimulus-response associations over stimulus-response-reward goal-directed action [4, 59].

To inform how serotonin might be engaged (i.e. released or inhibited) as reversal learning ensues, the following study in mice is informative. DRN 5-HT neuron activation tracked both positive and negative prediction errors during reversal learning [7]. These signals were qualitatively similar to dopaminergic prediction error signalling but differed in their time course: dopaminergic responses to cues were more quickly established and withdrawn. The authors posited it would follow that as cues result in more positive outcomes, dopaminergic signalling would be favoured temporarily thus invigorating behaviour, and when more negative outcomes emerge (during reversal, for instance) serotonergic signalling would be favoured instead, consequently promoting behavioural inhibition [7]. The contribution of dopaminergic versus serotonergic signalling would differ across valence conditions, and this framework, derived from an instrumental paradigm, can be extended to Pavlovian reversal as well.

Consideration of the influence of serotonin on specific amygdala sub-nuclei in rodents may also inform our human conditioning findings. The central nucleus of the amygdala (CeA) is the major source of output from the amygdala and its downstream projections ultimately produce defence responses such as perspiration in humans and freezing in rodents [122]. Critically, cells expressing 5-HT2A receptors in the CeA are differentially engaged by innate versus learned threats [72]. Inhibition of these 5-HT2A-expressing cells upregulates innate threat responses in mice and downregulates learned threat responses [72]. This is remarkably congruent with our observation that reducing serotonin function potentiated conditioning to innate threats, on the one hand, and findings from previous studies that reduction of serotonin signalling attenuates threat conditioning to learned (neutral) cues [69, 70]. The implication is that threat (here faces) normally releases 5-HT onto excitatory 5-HT2 receptors of the amygdala system that normally restrains innate aversion and promotes conditioning. Thus, ATD disinhibited innate responses (SCRs) to the face CS+, resulting in what appeared to be greater initial conditioning but may actually reflect larger autonomic responses that do not consolidate to form an associative memory. These divergent results may inform therapeutic, and possibly adverse, effects of serotonin modulating drugs. Indeed, risperidone (amongst other things a 5-HT2 receptor antagonist) exacerbated responses to innate threats and alleviated threat responses to previously neutral cues [72]. Furthermore, humans with selective damage to the basolateral amygdala (BLA), with the CeA preserved, showed hypervigilant responses to fearful faces (innate threat), which was interpreted as the removal of an inhibitory influence of the BLA over the CeA [123]. Indeed, the BLA receives particularly rich serotonergic innervation [57], which, in conjunction with the role of serotonin in the CeA for innate threats may be important for understanding our results.

### Limitations

As in other human ATD studies we did not measure serotonin directly, and instead used a widely accepted proxy measurement [75]. Whilst some have criticised ATD as a technique for studying serotonin in particular [124], the method has been robustly defended [77, 125]. Critically, the present findings align with deficits following profound neurotoxic serotonin depletion in both rats [52] and marmoset monkeys [54, 55, 113]. Our results build upon other studies, for instance on ‘waiting impulsivity’, that show parallel behavioural effects between neurotoxic depletions in experimental animals [126] and ATD in healthy humans [116], thus further bolstering the validity of ATD for studying serotonin.

Other limitations include the sample size of Experiment 2, which was relatively small. There were slight differences in Experiments 1 and 2 with respect to the inclusion criteria and general procedures, including different amino acid mixtures (see Supplementary Information), as they were conducted independently from one another. Experiment 2, moreover, contained only one reversal, whereas Experiment 1 incorporated multiple reversals; however, as a result, habituation of SCR, which can often occur in later phases of a threat conditioning experiment, may have been avoided [65, 127].

Whilst we did not apply psychophysiological modelling to the SCR data, which has the potential to increase effect sizes and has gained traction as an analysis approach [80], we observed large effect sizes nonetheless with base-to-peak scoring of SCR. The method used here, furthermore, was best at distinguishing different phases of an experiment [80], which is consistent with our primary aim of determining whether ATD affected acquisition, reversal, or both.

We did not apply computational modelling to the Pavlovian data, which in future efforts could reveal how serotonin influences associative learning dynamics in finer detail [13, 81, 117]. The feedback structure of the instrumental task, furthermore, was not conducive to standard reinforcement learning models, and could be modified in future studies to this end.

ATD can affect early processing in the auditory cortex [128], and our effects in Experiment 1 were seen in conditions of salient auditory feedback. Whilst the contribution of serotonin in sensory versus frontal areas (e.g. OFC) cannot be determined from the present data, auditory processing was not required in Experiment 2, yet reversal impairments were observed. More generally, the OFC is proposed to represent task states (e.g. given the current state, is choice A or B best?) and the extent of involvement of sensory areas is likely to depend on whether the state can be inferred from perceptual information (observable) or unobservable information (e.g. from working memory) [129].

## Conclusions

We provide evidence of human reversal learning impairments following serotonin depletion, in both the instrumental and Pavlovian domains, across two independent experiments. Deficits in both domains were underscored by significant correlations showing that a greater extent of depletion, as assessed by plasma samples, was associated with more pronounced reversal impairments. Strikingly, the results align with data from neurotoxic serotonin depletion in experimental animals [52, 54, 55, 113], stress induction in humans [117] and rats [56], and individuals with OCD [11].

That serotonin depletion impaired these fundamental learning processes pervasive in daily life highlights a failure mode that could lead to significant distress and impairment. The reversal deficits presented, furthermore, indicate how serotonergic dysfunction could impede the ability to engage in cognitive behavioural therapies. The present results therefore advance knowledge on the neurochemical basis of flexible Pavlovian and instrumental learning, which has implications for the understanding and treatment of numerous clinical conditions including OCD.

## Supplementary information

Supplementary Information

Supplementary Information
