# Supplementary material for: Serotonin depletion impairs both Pavlovian and instrumental reversal learning in healthy humans
Source: Mol Psychiatry. 2021 Aug 24;26(12):7200–10. doi: 10.1038/s41380-021-01240-9 (PMC8873011; doi:10.1038/s41380-021-01240-9)
Supplement: Supplementary file 1 — Supplementary Information [file 41380_2021_1240_MOESM1_ESM.docx]

**Supplemental Information**

**Methods: acute tryptophan depletion**

Tryptophan, the precursor required for serotonin synthesis, is an essential amino acid: it cannot be produced by the body and therefore must be obtained from the diet. Acute tryptophan depletion (ATD) is therefore a dietary technique for the study of serotonin, which rapidly decreases serotonin function (Bel & Artigas, 1996; Biggio et al., 1974; Crockett et al., 2012a; Nishizawa et al. 1997). Plasma samples were analysed using high performance liquid chromatography (HPLC) as in Crockett et al. (2013) and Passamonti et al. (2012). Depletion was indexed using the ratio of tryptophan to large neutral amino acids (TRP:LNAA), a widely accepted proxy measurement thought to be most reflective of brain serotonin (Bell et al. 2005). The LNAAs were valine, methionine, isoleucine, leucine, tyrosine, phenylalanine, and tryptophan. In other words, it was measurements of these listed amino acids that were extracted from the blood samples collected.

The amino acid quantities employed in Experiment 1 were derived from Worbe et al. (2014) and were as follows. The depletion mixture contained 4.10g L-alanine, 3.70g L-arginine, 8.93g L-aspartic acid, 2.00g L-cystine, 2.40g glycine, 2.40g L-histidine, 6.00g L-isoleucine, 10.10g L-leucine, 6.70g L-lysine, 2.30g L- methionine, 4.30g L-phenylalanine, 9.20g L-proline, 5.20g L-serine, 4.90g L-threonine, 3.00g L-tyrosine, and 6.70g L-valine. The placebo mixture was identical but contained 5.20g of L-tryptophan. The mixtures were manufactured by metaX Institut fur Diatetik GmbH with flavouring included. These drinks were prepared by stirring in 500ml tap water.

The amino acid quantities employed in Experiment 2 were derived from Crockett et al. (2009) and were as follows. Tryptophan depletion: 4.10g L-alanine, 3.70g L-arginine, 2.00g L-cystine, 2.40g glycine, 2.40g L-histidine, 6.00g L-isoleucine, 10.10g L- leucine, 6.70g L-lysine, 2.30g L-methionine, 9.20g L-proline, 4.30g L-phenylalanine, 5.20g L-serine, 4.90g L-threonine, 5.20g L-tyrosine, and 6.70g L-valine. The placebo mixture was the same as above, plus 3.00g of L-tryptophan. For females, 20% reductions in the above quantities were used to account for lower body weight. The mixtures were manufactured by SHS International, Liverpool, UK. These drinks were prepared by stirring the mixtures and adding lemon-lime flavouring into 200ml tap water.

**Methods: statistics**

The Greenhouse-Geisser correction was used where applicable, in designs with within-subjects factors, to correct for violation of the sphericity assumption as determined by Mauchly’s test. Degrees of freedom were adjusted for t-tests when Levene’s Test for equality of variances was violated.

**Methods: participants**

Exclusion criteria for Experiment 1 also encompassed neurological disorders; pregnancy; past use of neurological, psychiatric, or endocrine medication (including St John's wort); or current use of any regular medication besides contraceptive pills. The cut-offs for drug use were smoking more than five cigarettes per day, regular consumption of more than 38 UK units (380 ml) of alcohol per week, cannabis use more than once per month, and the lifetime use of recreational drugs besides cannabis more than five times. Other medical exclusion criteria: cardiac or circulation issues, respiratory problems including asthma; gastrointestinal, renal, or thyroid conditions; bleeding disorders, diabetes and head injury. Other exclusion criteria for Experiment 2 included medication use, a history of neurological, cardiac, gastrointestinal, hepatic, pulmonary, or renal disorders.

**Results: Impact of correct and erroneous responses on subsequent choices**

To determine whether feedback from correct or erroneous responses impacted subsequent actions, a win-stay/lose-shift analysis was performed. In other words, this analysis served to ascertain whether ATD affected whether participants obeyed the feedback received, by maintaining the same behavior after receiving positive feedback or shifting action after an incorrect response, both of which are adaptive under a deterministic reinforcement schedule. The number of wins on trials following a win was divided by the number of wins experienced, to yield a win-stay rate. Likewise, the number of wins on trials following a loss was divided by the number of losses experienced, to yield a lose-shift rate. For simplicity, all trials, including the first trial of each reversal phase, were analyzed.

ANOVA with serotonin status as between-subjects factor and valence (Reward-Punishment, Reward-Neutral, Punishment-Neutral, Neutral-Neutral) and behavior type (win-stay, lose-shift) as within-subjects factors yielded no main effect of serotonin status (F_(1,67)_ = .701, p = .405, η_p_^2^ = .010) nor any interactions with serotonin status (F < .830, p > .470, η_p_^2^ < .015, for all terms involving serotonin status). There was a significant effect of valence (F_(3,201)_ = 6.498, p = 3.23 × 10^-4^, η_p_^2^ = .088) and behavior type (F_(1,67)_ = 152.045, p = 6.753 × 10^-19^, η_p_^2^ = .694) as well as a valence × behavior type interaction (F_(3,201)_ = 8.291, p = 3.2 × 10^-5^, η_p_^2^ = .110). These findings indicate no specific effect of tryptophan depletion on reward or non-reward.

**Results: reaction times**

First, we examined whether ATD had an effect on the time window for valid responses as determined by the training phase (see Materials and methods). Independent samples t-test revealed that the time window to respond did not differ between the ATD and placebo groups (t_(67)_ = .957, p = .342, d = .231). Next, we conducted a repeated measures ANOVA with serotonin status (placebo, depletion) as between-subjects factor, valence (reward-punishment, reward-neutral, punishment-neutral, neutral-neutral) and block (acquisition, reversal 1, reversal 2, reversal 3) as within-subjects factors, which revealed significant effects of valence (F_(3,201)_ = 4.581, p = .004, η_p_^2^ = .064) and block (F_(3,201)_ = 5.064, p = .002, η_p_^2^ = .070), as well as a significant serotonin × block interaction (F_(3,201)_ = 3.394, p = .019, η_p_^2^ = .048). There was no main effect of serotonin status (F_(1,67)_ = 1.770, p = .188, η_p_^2^ = .026) nor was there a serotonin × valence interaction (F_(3,201)_ = 1.206, p = .309, η_p_^2^ = .018) or a serotonin × valence × block interaction (F_(7,469)_ = .655, p = .710, η_p_^2^ = .010). Follow-up paired t-tests indicated the serotonin × block interaction was driven by a decrease in reaction times in the placebo group from block 2 to block 3 (t_(33)_ = 3.968, p = 3.68 × 10^-4^, d = .681) and block 3 to block 4 (t_(33)_ = 2.991, p = .005, d = .513) that was not present in the ATD group (blocks 2 to 3: t_(34)_ = -.091, p = .928, d = -.015; blocks 2 to 4: t_(34)_ = .940, p = .354, d = .159).

A breakdown of how reaction times differed across valence conditions is outlined in Supplemental Table 4. Follow-up paired t-tests indicated that reaction times were greater in the neutral-neutral condition compared to the reward-neutral (t_(68)_ = 3.787, p = 3.26 × 10^-4^, d = .456) and punishment-neutral (t_(68)_ = -2.066, p = .043, d = -.249) conditions but not the reward-punishment (t_(68)_ = -1.499, p = .139, d = -.180) condition. Reaction times in the reward-punishment condition were greater than in the reward-neutral condition (t_(68)_ = 2.213, p = .030, d = .266), but did not differ from the punishment-neutral condition (t_(68)_ = -.646, p = .520, d = -.078). There was no difference in reaction time between the reward-neutral and punishment-neutral conditions (t_(68)_ = 1.508, p = .136, d = .181).

Follow-up paired t-tests also revealed that reaction times did not differ between blocks 1 and 2 regardless of valence or serotonin status (t_(68)_ = .311, p = .757, d = .037), but reaction times decreased after block 2 (block 2 to block 3: t_(68)_ = 2.450, p = .017, d = .295; block 2 to block 4: t_(68)_ = 2.859, p = .006, d = .344). Reaction times did not differ between blocks 3 and 4 (t_(68)_ = -.242, p = .809, d = -.029).

**Results: accuracy of finger and hand selection**

Repeated measures ANOVA was conducted on trials to criterion (with factors of serotonin status, valence, and block) to assess the accuracy of finger and hand selection. There was no main effect of ATD on accuracy of finger selection (F_(1,67)_ = 2.665, p = .107, η_p_^2^ = .038) nor were there interactions of serotonin × valence (F_(3,201)_ = .615, p = .606, η_p_^2^ = ..009), serotonin × block (F_(3,201)_ = 1.868, p = .136, η_p_^2^ = .027), or serotonin × valence × block (F_(7,475)_ = .767, p = .617, η_p_^2^ = .011). There was also no main effect of ATD on accuracy of hand selection (F_(1,67)_ = 3.930, p = .052, η_p_^2^ = .055) nor were there interactions of serotonin × valence (F_(3,201)_ = 1.113, p = .345, η_p_^2^ = .016), serotonin × block (F_(3,201)_ = 1.937, p = .125, η_p_^2^ = .028), or serotonin × valence × block (F_(7,466)_ = .425, p = .886, η_p_^2^ = .006).

**Results: order of valence conditions**

Whilst the order of valence conditions was randomly generated for each participant, we conducted ANOVAs to assess whether there were order effects on the main behavioural outcome measure, trials to criterion. For simplicity, ANOVAs were conducted without serotonin status as a factor and were performed separately for each valence condition. The first ANOVA addressed the question: did the placement of the reward-punishment condition in the sequence of four valence runs influence performance? ANOVA with order (1, 2, 3, or 4) as between-subjects factor and block (1, 2, 3, or 4) as within-subjects factor revealed there was no main effect of order on trials to criterion in the reward-punishment condition (F_(1,65)_ = 1.846, p = .148, η_p_^2^ = .078) nor was there an order × block interaction (F_(9,195)_ = 1.197, p = .299, η_p_^2^ = .052). ANOVA with the same factors was conducted for the reward-neutral condition and showed there was no main effect of order (F_(1,65)_ = 2.388, p = .077, η_p_^2^ = .099) on trials to criterion, nor was there an order × block interaction (F_(9,195)_ = 1.492, p = .153, η_p_^2^ = .064). The results of these two ANOVAs indicate the core results of the instrumental reversal experiment – which appeared in the reward-punishment and reward-neutral conditions – were not driven by order effects.

For the punishment-neutral condition, ANOVA revealed a main effect of order (F_(1,65)_ = 5.036, p = .003, η_p_^2^ = .189) but no order × block interaction (F_(9,195)_ = 1.779, p = .074, η_p_^2^ = .076). In the neutral-neutral condition, ANOVA indicated a main effect of order (F_(1,65)_ = 3.327, p = .025, η_p_^2^ = .133) and no order × block interaction (F_(9,195)_ = 1.209, p = .292, η_p_^2^ = .053). The significant main effect of order in the punishment-neutral condition and the neutral-neutral condition were each followed up with an additional ANOVA adding serotonin status back into the model as an additional between-subjects factor, to ensure the order effects did not interact with serotonin status. Indeed, there was no serotonin × order interaction in the punishment-neutral condition (F_(3,61)_ = .265, p = .850, η_p_^2^ = .013) nor in the neutral-neutral condition (F_(3,61)_ = .868, p = .463, η_p_^2^ = .041).

**Supplemental Table 1.**

|  | **Placebo**  **Mean (SD)** | **Depletion**  **Mean (SD)** | **t(df)** | **p** |
| --- | --- | --- | --- | --- |
| Age | 24.24 (5.684) | 24.31 (4.788) | .063 (67) | .95 |
| Years of education | 16.97 (2.181) | 17.34 (2.376) | .678 (67) | .5 |
| BDI-II | 4.76 (4.335) | 3.80 (3.701) | -.995 (67) | .323 |
| OCI-R | 8.21 (7.543) | 7.09 (7.172) | -.632 (67) | .529 |
| BIS | 70.41 (4.150) | 71.09 (3.673) | .715 (67) | .477 |

Demographics and questionnaire measures for Experiment 1. BDI-II = Beck Depression Inventory, version II (Beck et al. 1996); OCI-R = Obsessive Compulsive Inventory (Foa et al. 2002); BIS = Barratt Impulsiveness Scale (Patton et al. 1995). SD = standard deviation.

**Supplemental Table 2.**

Demographics and questionnaire measures for Experiment 2. BDI-II = Beck Depression Inventory, version II (Beck et al. 1996); BIS = Barratt Impulsiveness Scale (Patton et al. 1995). SD = standard deviation. Age was unavailable for one participant in the placebo condition. In the depletion condition, age was unavailable for two participants, and years of education were unavailable for three participants.

|  | **Placebo**  **Mean (SD)** | **Depletion**  **Mean (SD)** | **t(df)** | **p** |
| --- | --- | --- | --- | --- |
| Age | 25.73 (3.80) | 25.21 (2.860) | -.694 (24) | .495 |
| Years of education | 18.33 (1.923) | 18.46 (2.727) | -.135 (23) | .894 |
| BDI-II | 3.42 (3.423) | 4.06 (4.739) | -.400 (26) | .693 |
| BIS | 55.33 (6.827) | 59.81 (8.272) | -1.524 (26) | .139 |

**Supplemental Table 3.**

Instrumental performance (Experiment 1) by valence and block.

|  | **# of subjects not meeting criterion** | | | **Mean (SD)**  **trials to criterion** | | | |
| --- | --- | --- | --- | --- | --- | --- | --- |
|  | **Groups collapsed** | **Placebo** | **ATD** | **Placebo** | **ATD** | **t(df)** | **p** |
| N-N: acquisition | 9 | 2 | 7 | 7.5 (5.3) | 8.8 (7.0) | .89(64) | .376 |
| N-N: reversal 1 | 14 | 4 | 10 | 8.4 (5.7) | 10.1 (7.3) | 1.1(64) | .297 |
| N-N: reversal 2 | 8 | 4 | 4 | 7.8 (6.1) | 8.7 (6.0) | .57(67) | .569 |
| N-N: reversal 3 | 7 | 3 | 4 | 8.8 (5.4) | 8.1 (5.8) | -.53(67) | .601 |
| P-N: acquisition | 15 | 8 | 7 | 9.9 (6.9) | 8.7 (6.8) | -.69(67) | .494 |
| P-N: reversal 1 | 11 | 5 | 6 | 7.8 (6.0) | 9.9 (6.3) | 1.4(67) | .155 |
| P-N: reversal 2 | 17 | 9 | 8 | 9.8 (7.4) | 8.6 (7.3) | -.51(67) | .610 |
| P-N: reversal 3 | 11 | 2 | 9 | 8.0 (5.5) | 10.0 (6.7) | 1.3(67) | .182 |
| R-N: acquisition | 13 | 8 | 5 | 9.3 (7.1) | 8.8 (6.2) | -.325(67) | .746 |
| R-N: reversal 1 | 13 | 3 | 10 | 8.2 (5.9) | 11.5 (6.7) | 2.1(67) | .038 |
| R-N: reversal 2 | 14 | 3 | 11 | 6.4 (5.5) | 10.3 (7.8) | 2.4(61) | .019 |
| R-N: reversal 3 | 13 | 6 | 7 | 8.3 (7.0) | 8.1 (7.0) | -.12(67) | .902 |
| R-P: acquisition | 4 | 1 | 3 | 5.9 (5.23) | 7.4 (5.8) | 1.1(67) | .269 |
| R-P: reversal 1 | 3 | 1 | 2 | 6.4 (4.7) | 5.8 (5.0) | -.53(67) | .599 |
| R-P: reversal 2 | 6 | 0 | 6 | 5.7 (4.1) | 8.7 (6.3) | 2.3(59) | .026 |
| R-P: reversal 3 | 4 | 1 | 3 | 4.9 (4.4) | 6.3 (5.9) | 1.1(67) | .277 |

N-N = neutral-neutral block; P-N = punishment-neutral block; R-N = reward-neutral block; R-P = reward-punishment block

**Supplemental Table 4.** Reaction time by valence condition, regardless of serotonin status

|  | RT (ms) mean | RT SD |
| --- | --- | --- |
| Neutral-neutral | 512.380 | 67.556 |
| Punishment-neutral | 503.879 | 65.884 |
| Reward-neutral | 498.965 | 61.190 |
| Reward-punishment | 506.220 | 63.500 |

RT = reaction time; ms = milliseconds; SD = standard deviation

**Supplemental Figure 1.** Experiment 2 task schematic, taken from Apergis-Schoute et al. (2017) with permission. Face stimuli used with permission from Paul Ekman, PhD/Paul Ekman, LLC.


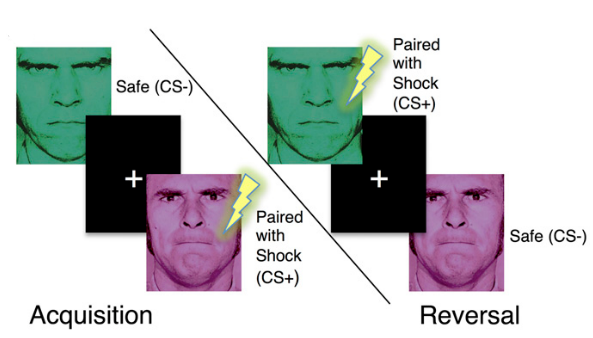


**Supplementary Figure 2.** Feedback icons for the task. Left to right: correct, incorrect, too late.


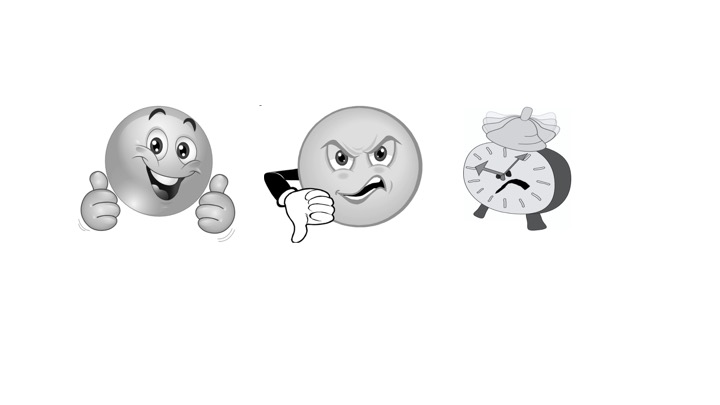


**References**

Crockett MJ, Clark L, Robbins TW (2009) Reconciling the role of serotonin in behavioral inhibition and aversion: acute tryptophan depletion abolishes punishment-induced inhibition in humans. J Neurosci 29:11993–11999. doi: 10.1523/JNEUROSCI.2513-09.2009

Zhang Z, Manson KF, Schiller D, Levy I (2014) Impaired associative learning with food rewards in obese women. Curr Biol 24:1731–1736. doi: 10.1016/j.cub.2014.05.075
