# Supplementary figures and images for: Serotonin depletion impairs both Pavlovian and instrumental reversal learning in healthy humans
Source: Mol Psychiatry. 2021 Aug 24;26(12):7200–10. doi: 10.1038/s41380-021-01240-9 (PMC8873011; doi:10.1038/s41380-021-01240-9)

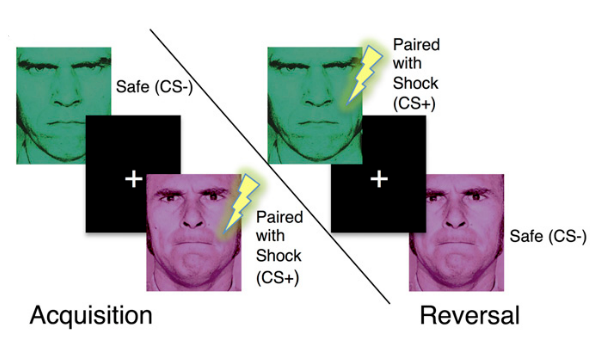

Supplement: Supplementary file 2 — Supplementary Information [file 41380_2021_1240_MOESM2_ESM.png]
